# Subacute Thyroiditis is Associated with HLA-B*18:01, -DRB1*01 and -C*04:01—The Significance of the New Molecular Background

**DOI:** 10.3390/jcm9020534

**Published:** 2020-02-16

**Authors:** Magdalena Stasiak, Bogusław Tymoniuk, Renata Michalak, Bartłomiej Stasiak, Marek L. Kowalski, Andrzej Lewiński

**Affiliations:** 1 Department of Endocrinology and Metabolic Diseases, Polish Mother’s Memorial Hospital Research Institute, 281/289 Rzgowska St., 93-338 Lodz, Poland; mstasiak33@gmail.com (M.S.); renatkamichalak@gmail.com (R.M.); 2 Department of Immunology, Rheumatology and Allergy, Medical University of Lodz, 251 Pomorska St, 92-213 Lodz, Poland; boguslaw.tymoniuk@gmail.com (B.T.); marek.kowalski@umed.lodz.pl (M.L.K.); 3 Institute of Information Technology, Lodz University of Technology, 215 Wolczanska St., 90-924 Lodz, Poland; bartlomiej.stasiak@p.lodz.pl; 4 Department of Endocrinology and Metabolic Diseases, Medical University of Lodz, 281/289 Rzgowska St., 93-338 Lodz, Poland

**Keywords:** HLA-B*35, HLA-B*18:01, HLA-DRB1*01, HLA-C*04:01, subacute thyroiditis

## Abstract

Subacute thyroiditis (SAT) is a thyroid inflammatory disease whose pathogenesis is still not completely defined. Previous viral infection is considered to be a triggering factor in genetically predisposed individuals. In about 70% of patients, susceptibility to SAT is associated with the HLA-B*35 allele. The correlation between SAT and other human leukocyte antigens (HLA) has not yet been unequivocally demonstrated and the genetic background is still unknown in about 30% of patients. The purpose of our study was to perform HLA genotyping using a next-generation sequencing method, to find out whether alleles other than HLA-B*35 are correlated with SAT morbidity. *HLA-A, -B, -C, -DQB1, -DRB1* were genotyped using a next-generation sequencing method in 1083 subjects, including 60 SAT patients and 1023 healthy controls. Among 60 patients diagnosed with SAT, 81.7% of subjects were identified as having allele HLA-B*35, 23.3% had HLA-B*18:01, 28.3% had HLA-DRB1*01 and 75.5% had HLA-C*04:01. These alleles occurred in the control group at frequencies of 10.2%, 7.2%, 12.9% and 12.5%, respectively. The differences were statistically significant, with *p* < 0.05. In addition to its previously described relationship with HLA-B*35, genetic susceptibility to SAT was associated with the presence of HLA-B*18:01, DRB1*01 and C*04:01. The alleles HLA-B*18:01 and DRB1*01 were independent SAT risk factors. The assessment of these four alleles allows the confirmation of genetic predisposition in almost all patients with SAT.

## 1. Introduction

Subacute thyroiditis (SAT) (also called granulomatous thyroiditis, giant cell thyroiditis or de Quervain’s thyroiditis) is a thyroid inflammatory disease of still-uncertain pathogenesis. However, previous viral infection (occurring approximately 2–6 weeks beforehand) is considered to be a triggering factor in genetically predisposed individuals. The prevalence of SAT is the highest in middle aged women, and female patients account for 75%–80% of individuals with the disease [1,2]. The most common presentation is anterior neck pain radiating ipsilaterally up to the jaw and ear, and down to the upper part of the chest [2,3,4]. However, cases of SAT without pain are more and more often described [2,5,6,7]. Fever is often present, with temperatures frequently reaching over 39°C, rising especially at night. Patients usually complain of asthenia and malaise, and some symptoms of thyrotoxicosis may be present. Laboratory findings include a characteristically high erythrocyte sedimentation rate (ESR). The C reactive protein (CRP) level is also elevated and laboratory markers of hyperthyroidism are often present, but the level of thyroid antibodies is normal in most patients [2,3,4]. The ultrasound (US) features of SAT include hypoechoic and heterogeneous areas with blurred margins, and poor vascularization according to the Color Doppler [8,9]. 

Although the natural course of the disease is self-limiting, patients frequently suffer from severe symptoms that make normal functioning impossible, sometimes for months.

Susceptibility to the disease is associated with the occurrence of certain types of human leukocyte antigen (HLA). In 1975, Nyulassy et al. [10] first reported that the frequency of *HLA-B35* was significantly increased in patients with SAT. Since then, a prominent correlation between SAT and *HLA-B35* has been confirmed by many authors, in several different populations [11,12,13,14,15,16,17,18,19]. Some authors have suggested other possible associations between HLA and SAT. All of them used much older methods, especially those of a serological nature, with which accuracy is significantly lower than with the high-resolution method used in our study. Moreover, the symbols of the particular alleles assessed by the previously applied methods differ from those currently used, and some antigens previously denoted by one symbol are separated as several individual alleles when assessed by the high-resolution method. Using the serological method, Ohsako et al. [20] demonstrated an increased occurrence of HLA-Bw67 and HLA-Cw3 in 56 Japanese patients with SAT, compared to in 238 healthy people. Unfortunately, no other authors have confirmed the association of SAT with HLA-B*67 (current nomenclature) and the potential correlation with HLA-Cw3 has been described again only once—also in a Japanese population [21]. A few authors have also suggested a possible correlation between SAT and HLA-Cw4 [15]. Weak associations between SAT and HLA-Dw1 [22] (current nomenclature—HLA-DRB1*01) and HLA-DRw8 (current nomenclature—HLA-DRB1*08) [14] have been observed, but the differences were not statistically significant. A statistically significant higher frequency of HLA-DRw8 in SAT has been reported only once [20]. 

The presence of *HLA-B35* has been observed in about 70% of SAT patients [12,20,21], but the other possible associations suggested so far have not been definitively confirmed. However, it seems doubtful that in *HLA-B35*-negative patients, SAT would occur in the absence of any genetic susceptibility. Therefore, there was a need to re-analyze HLA profiles with methods more modern than those previously used, and to compare the SAT group to a significantly larger control group than had been used in studies described so far.

The purpose of the study was to re-evaluate class I and class II major histocompatibility complex (MHC) antigens in 60 SAT patients and 1023 healthy controls, and to find out whether antigens other than *HLA-B35* were correlated with SAT morbidity.

## 2. Materials and Methods

### 2.1. HLA Typing Procedures

High-resolution HLA typing was performed in 1023 potential healthy Polish hematopoietic stem cell donors with no medical history of thyroid disease, and in 60 patients who were diagnosed with SAT between 2003 and 2019 in the Department of Endocrinology and Metabolic Diseases, Polish Mother’s Memorial Hospital—Research Institute, Lodz, Poland. HLA-A, -B, -C, -DQB1 and -DRB1 were genotyped using a next-generation sequencing method on the Illumina platform (Illumina, USA). Sequencing-based HLA typing of the HLA genes -A, -B, -C, -DQB1 and -DRB1 was carried out in 96-well format within a semi-automated workflow using MiaFora Flex5 typing kits (Immucor, USA). Long-range PCR amplification of five HLA loci was performed on DNA extracted from blood samples. Results of sequencing were analyzed using the MiaFora NGS software. Data were considered sufficient whenever the coverage reached 40 and the number of cReads exceeded 50 000. The sequencing included extensive coverage of the HLA genome, especially with respect to the five loci.

### 2.2. Statistical Analysis

Phenotype frequencies were reported as absolute values and in percentages. The statistical significance of the differences between groups was evaluated by the χ2 test and by binomial logistic regression analysis, with p values <0.05 considered significant. The statistical analysis was carried out using the Statistica v 13.1 software (Statsoft Polska, Poland). 

### 2.3. Inclusion Criteria

The diagnosis of SAT was based on the diagnostic criteria recently proposed by our team [23]. These criteria are as follows: elevation of ESR (or at least CRP) plus hypoechoic area/areas with blurred margins and decreased vascularization according to ultrasonography; cytological confirmation of SAT, or at least cytological exclusion of malignancy; plus at least one of the following: hard thyroid swelling and/or pain and tenderness of the thyroid gland/lobe, elevation of serum free thyroxine (FT4) and suppression of thyroid stimulating hormone (TSH), and decreased radioiodine uptake (RAIU). 

### 2.4. Biochemical and Cytological Procedures

Serum levels of TSH and FT4 were measured by the electrochemiluminescence immunoassay (ECLIA) with the Cobas e601 analyzer (Roche Diagnostics, USA), ESR was determined with the Ves-Matic Cube 30 (Diesse, Italy) and CRP was determined using the VITROS^®^ 4600 Chemistry System (Ortho Clinical Diagnostics, USA). Ultrasound examinations (US) were performed in every patient, using a 7–14 MHz linear transducer (Toshiba Aplio XG; Toshiba, Japan). Fine needle aspiration biopsies (FNAB) were performed in all SAT patients using a 23-gauge needle. Smears were cytologically evaluated, and the presence of multinucleated giant cells together with mononucleated macrophages—and of follicular epithelial cells against an acute and chronic inflammatory dirty background (comprising of cellular debris and mixed inflammatory cells)—were considered to be results typical of SAT. 

### 2.5. Ethics Procedures

Informed consent for all performed procedures was obtained from all of the patients after a full explanation of the purpose and nature of all procedures used.

The study was approved by the Ethics Committee of the Polish Mother’s Memorial Hospital—Research Institute, Lodz, Poland (Project identification code—22/2016). 

## 3. Results

**Figure 1:**
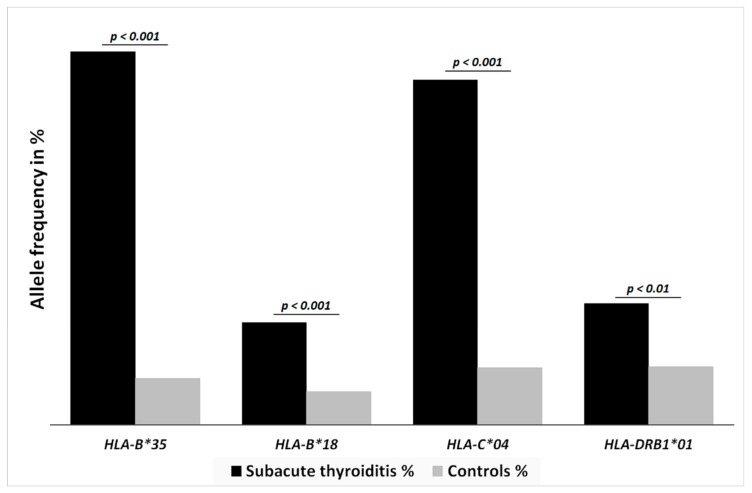
Black and gray columns show the haplotype frequencies in SAT patients and healthy controls, respectively. SAT was significantly associated with HLA-B*35 (*p* < 0.0001), HLA-B*18:01 (*p* = 0.0001), HLA-C*04:01 (*p* < 0.0001) and HLA-DRB1*01 (*p* = 0.007).

The age, gender and race of each patient included in the study are presented in Table S1. Among 60 patients diagnosed with SAT, 49 (81.7%) subjects were identified as having HLA-B*35, 14 (23.3%) with HLA-B*18:01, 17 (28.3%) with HLA-DRB1*01, 45 (75.5%) with HLA-C*04:01, seven (11.7%) with HLA-C*03, and four (6.7%) with HLA-DRB1*08 (Table 1). All of the results for allelic specificity are presented in Table 1, but the results for the same antigen but different allelic specificity were counted together, so as to enable comparison with data in the literature, and to present the association with SAT as clearly as possible (Figure 1). Statistically significant differences with respect to the healthy control group of 1023 subjects were reported; in the control group, only 104 (10.2%) were genotyped with HLA-B*35, 74 (7.2%) with HLA-B*18:01, 132 (12.9%) with HLA-DRB1*01, and 128 (12.5%) with HLA-C*04:01 (Figure 1). Combinations of two, three or four alleles were present in 30, 16 and two cases, respectively.

No statistically significant differences between the SAT group and controls were found with respect to the frequency of other HLA phenotypes, including those previously suggested: HLA-C*03 and -DRB1*08. We did not find any case of HLA-B*67 in the SAT patients or in the control group (due to large amount of statistical data regarding all analyzed alleles, the negative results are presented in Table 1 only for these two alleles, as they had been previously suggested).

## 4. Discussion

Subacute thyroiditis is a rare disease but its incidence is increasing. Many clinical symptoms of SAT are not characteristic, and diagnosis is often delayed, with many previous misdiagnoses and mistreatments being reported [24]. The data in the literature indicate that the occurrence of SAT is associated with genetic predispositions encoded in the sixth chromosome fragment responsible for the HLA tissue compatibility antigen system. A prominent correlation between SAT and HLA-B*35 has been widely described [11,12,13,14,15,16,17,18,19]. All of the previously published reports have demonstrated correlations either by comparison with much smaller control groups than ours, or only in individual cases with no data against which to compare [11,12,13,14,15,16,17,18,19]. The frequency of HLA-B*35 in SAT patients varied from 67 to 72.5% [12,20,21]. We compared our 60 SAT patients with 1023 healthy subjects, demonstrating an even stronger association, with the frequency exceeding 81% in SAT subjects as compared to being only 10.2% in the control group.

Obviously, it is to be expected that HLA-B*35-negative SAT patients also have some other HLA-related genetic susceptibility. Rubin and Guay [16] suggested a possible association between SAT and HLA-Cw4 in identical twins. This correlation remained questionable because it is difficult to draw any conclusions regarding the HLA-related background of any disease based on results obtained in identical twins. Our study, carried out in a large cohort with the application of the high-resolution HLA typing method, confirmed such correlation and revealed the strength of it, since we demonstrated the presence of HLA-C*04:01 in 75.5% of the SAT patients, as compared to in 12.5% of the control group. This finding has not been shown before, although it should have been expected, because HLA-C*04:01 is in linkage disequilibrium with HLA-B*35:01/02/03 (i.e., these two alleles commonly occur together due to the close location of their loci; their association is well-described [25]). Therefore, the occurrence of the HLA-B*35 and HLA-C*04:01 alleles is a marker of genetic susceptibility for SAT. However, HLA-C*04:01 alone cannot be considered as an independent SAT risk factor due to its linkage with HLA-B*35. No such linkage has been described for HLA-B*18:01 or -DRB*1:01, so these alleles should be considered completely independent SAT risk factors.

Ohsako et al. [20] reported an increased occurrence of HLA-Cw3 in 56 Japanese patients with SAT, as compared to in 238 healthy Japanese people. Kobayashi et al. [21] observed a high frequency (65.2%) of HLA-Cw3 in Japanese patients with SAT, but they did not compare that group to the general population. This relationship was not confirmed in our study, since we did not observe an increased frequency of HLA-C*03 in the SAT group. Such a result is to be expected, because—according to the most current sources—in populations other than Asian, there is no similar linkage disequilibrium of HLA-B*35 and -C*03 [26]. Therefore, in our exclusively Caucasian cohort, we did not find a similar correlation. This discrepancy in the obtained results may also be a consequence of the differences in the sizes of the studied cohorts, because while the numbers of patients in the SAT groups were similar, our control group was over four times larger than that of Ohsako et al. [20]. However, it seems most probable that the inconsistency resulted from having completely different study populations (Caucasians vs. Asians—also different in genetic terms).

The most important finding of the present study is the demonstration of the correlation between the occurrence of SAT, and HLA-B*18:01 and HLA-DRB1*01. To our best knowledge, our study is the first published showing the significance of HLA-B*18:01 and HLA-DRB1*01 in the pathogenesis of SAT. It should be noted that HLA-DRB1*01 belongs to class II of the MHC group, and therefore constitutes the only class I-independent SAT genetic marker. The independent association of HLA-B*18:01 and SAT is supported by our recent study demonstrating differences in the sonographic patterns of SAT associated with either -B*35 or -B*18:01 [27]. Additionally, the simultaneous presence of HLA-B*18:01 and -B*35 was demonstrated to increase the risk of SAT recurrence [28]. Thus, HLA-B*18:01 seems to modify the clinical features of SAT depending on whether it occurs alone or with *-B35*. The new data regarding the influence of SAT-risk HLA alleles on the clinical course of the disease may facilitate diagnosis and treatment. In some patients, the SAT US image may be challenging to evaluate, as—for example—the typical US pattern and vascularization in SAT is sometimes similar to that of the early phase of chronic autoimmune thyroiditis [29]. In HLA-B*18:01 carriers, the SAT US pattern may be highly misleading, largely resembling a large thyroid tumor [27]. Thus, the knowledge of the association between HLA and SAT US images can be useful in clinical practice. Awareness of the correlation between HLA and the risk of SAT recurrence seems even more important for disease management [28]. The details of the clinical courses of other HLA-related thyroid diseases were also demonstrated to depend on HLA antigens. Vita et al. [30] reported that HLA haplotype influenced the onset age and the severity of hyperthyroidism in Graves’ disease. Similarly, we have also observed correlation with onset age, with carriers of HLA-B*18:01 and -B*35 being significantly older at SAT onset (unpublished data). The strong influence of HLA-B*18:01 on the clinical course—i.e., on the US pattern, the recurrence risk and the onset age in SAT—is clearly visible [27,28]. Demonstrating the importance of HLA-B*18:01 and HLA-DRB1*01 complements the existing gap in the knowledge regarding the pathogenesis of, and genetic susceptibility to, SAT. The presence of HLA-B*18:01 was found in 23.3% of our patients with SAT, as compared to in 7.2% of the control group, while HLA-DRB1*01 occurred in 28.3% of SAT patients vs. 12.9% of controls. Owing to the finding of these new relationships between SAT, and HLA-B*18:01 and HLA-DRB1*01; and to the finding of such a strong correlation with HLA-C*04:01; the determination of the genetic background of SAT has become possible in virtually all patients. It is probable that some other, less prominent correlations are present in other populations, mainly in Asian ones, where other alleles may be in linkage disequilibrium with *HLA-B35*. HLA-B*18:01 and HLA-DRB1*01, being unassociated with HLA-B*35, are genetic risk factors which seem to be population-independent SAT markers. 

We did not confirm the association of SAT with any of the other previously suspected antigens, such as HLA-Bw67 [19] and HLA-DRw8, as no statistically significant differences were found in the occurrence of these antigens between our SAT group and controls.

## 5. Conclusions

In conclusion, our results obtained for a large healthy Polish cohort and SAT patients provide the finding that HLA-B*18:01 and HLA-DRB1*01 represent independent SAT risk alleles. Moreover, the HLA-B*35 and HLA-C*04:01 alleles were proven to be markers of genetic susceptibility to SAT, regardless of whether both of these antigens are present, or if only one of them is detected. Thus, our data definitely question the negative predictive value of the absence of HLA-B*35 in subjects with SAT or at risk of SAT. In fact, according to our data, a diagnosis of SAT or susceptibility to SAT can be confirmed genetically, based on the presence of any of the four HLA alleles that have been proven to be SAT-related by the current work. The results of the present study have filled in some of the missing elements of knowledge regarding the pathogenesis of SAT, and they may, therefore, form the basis for the development of a relatively simple genetic test, the use of which will facilitates and speeds up SAT diagnosis. Moreover—as proven by our research group—the presence of these new high-risk alleles (mainly HLA-B*18:01) has a great impact on the clinical course of SAT. 

## Figures and Tables

**Table 1:** The prevalence of human leukocyte antigen (HLA) alleles in subacute thyroiditis (SAT) patients and the control group.

HLA Allele	Subacute Thyroiditis % (No of Patients)	Healthy Controls % (No of Patients)	*p*-Value
HLA-B*35:01	38.8 (23)	5.2 (53)	*p < 0.0001*
HLA-B*35:02	11.7 (7)	1.2 (12)	*p < 0.0001*
HLA-B*35:03	35 (21)	3.4 (35)	*p < 0.0001*
HLA-B*35:08	3.3 (2)	0.4 (4)	*p < 0.0367*
HLA-B*35:01/02/03/08	81.7 (49)	10.2 (104)	*p < 0.0001*
HLA-B*18:01	23.3 (14)	7.2 (74)	*p = 0.0001*
HLA-C*04:01	75.5 (45)	12.5 (128)	*p < 0.0001*
HLA-DRB1*01:01	25 (15)	12.0 (130)	*p = 0.0126*
HLA-DRB1*01:02	1.7 (1)	0.1 (1)	*p = 0.2286*
HLA-DRB1*01:03	1.7 (1)	0.1 (1)	*p = 0.2286*
HLA-DRB1*01:01/02/03	28.3 (17)	12.9 (132)	*p = 0.007*
HLA-C*03	11.7 (7)	10 (102)	*p = 0.8386*
HLA-DRB1*08	6.7 (4)	3.4 (35)	*p = 0.3397*

## Supplementary Materials

The following are available online at https://www.mdpi.com/2077-0383/9/2/534/s1, Table S1: The age, gender and race of the patients included into the study.

## Abbreviations

CRP	C reactive protein
ECLIA	electrochemiluminescence immunoassay
ESR FT4	erythrocyte sedimentation rate free thyroxine
HLA	human leukocyte antigens
MHC	major histocompatibility complex
SAT TSH	subacute thyroiditis thyroid stimulating hormone
US	ultrasound
