# Supplementary material for: Subacute Thyroiditis is Associated with HLA-B*18:01, -DRB1*01 and -C*04:01—The Significance of the New Molecular Background
Source: J Clin Med. 2020 Feb 16;9(2):534. doi: 10.3390/jcm9020534 (PMC7074389; doi:10.3390/jcm9020534)
Supplement: Supplementary file 1 [file jcm-09-00534-s001.pdf]

Table S1: The age, gender and race of the patients included into the study.

Patient No.	Age	Gender	Race
**1.**	43	F	Caucasian
**2.**	35	M	Caucasian
**3.**	31	F	Caucasian
**4.**	28	F	Caucasian
**5.**	52	F	Caucasian
**6.**	48	M	Caucasian
**7.**	34	M	Caucasian
**8.**	36	F	Caucasian
**9.**	43	F	Caucasian
**10.**	31	F	Caucasian
**11.**	36	F	Caucasian
**12.**	51	F	Caucasian
**13.**	42	F	Caucasian
**14.**	41	F	Caucasian
**15.**	50	F	Caucasian
**16.**	50	M	Caucasian
**17.**	36	M	Caucasian
**18.**	36	F	Caucasian
**19.**	37	F	Caucasian
**20.**	37	F	Caucasian
**21.**	34	F	Caucasian
**22.**	40	M	Caucasian
**23.**	57	F	Caucasian
**24.**	31	F	Caucasian
**25.**	48	F	Caucasian
**26.**	63	F	Caucasian
**27.**	44	F	Caucasian
**28.**	38	F	Caucasian
**29.**	39	F	Caucasian
**30.**	35	F	Caucasian
**31.**	44	F	Caucasian
**32.**	58	F	Caucasian
**33.**	47	F	Caucasian
**34.**	33	F	Caucasian
**35.**	48	F	Caucasian
**36.**	47	F	Caucasian
**37.**	51	F	Caucasian
**38.**	50	F	Caucasian
**39.**	43	M	Caucasian

**40.**	42	F	Caucasian
**41.**	69	F	Caucasian
**42.**	45	M	Caucasian
**43.**	35	F	Caucasian
**44.**	36	F	Caucasian
**45.**	38	F	Caucasian
**46.**	56	F	Caucasian
**47.**	77	F	Caucasian
**48.**	65	F	Caucasian
**49.**	62	F	Caucasian
**50.**	58	F	Caucasian
**51.**	53	F	Caucasian
**52.**	45	F	Caucasian
**53.**	42	F	Caucasian
**54.**	40	M	Caucasian
**55.**	38	F	Caucasian
**56.**	30	F	Caucasian
**57.**	30	F	Caucasian
**58.**	45	F	Caucasian
**59.**	46	F	Caucasian
**60.**	65	F	Caucasian

Abbreviations: F, female; M, male.
